# Iron Status May Not Affect Amyotrophic Lateral Sclerosis: A Mendelian Randomization Study

**DOI:** 10.3389/fgene.2021.617245

**Published:** 2021-03-04

**Authors:** Jiahao Cai, Xiong Chen, Hongxuan Wang, Zixin Wei, Mei Li, Xiaoming Rong, Xiangpen Li, Ying Peng

**Affiliations:** ^1^Department of Neurology, Sun Yat-sen Memorial Hospital, Sun Yat-sen University, Guangzhou, China; ^2^Department of Urology, Sun Yat-sen Memorial Hospital, Sun Yat-sen University, Guangzhou, China; ^3^Department of Pulmonary and Critical Care Medicine, Sun Yat-sen Memorial Hospital, Sun Yat-sen University, Guangzhou, China; ^4^Guangdong Provincial Key Laboratory of Malignant Tumor Epigenetics and Gene Regulation, Sun Yat-sen Memorial Hospital, Sun Yat-sen University, Guangzhou, China

**Keywords:** iron, neurodegeneration, amyotrophic lateral sclerosis, Mendelian randomization, causality

## Abstract

**Background:**

Observational studies have shown an association of increased iron status with a higher risk of amyotrophic lateral sclerosis (ALS). Iron status might be a novel target for ALS prevention if a causal relationship exists. We aimed to reveal the causality between iron status and ALS incidence using a large two-sample Mendelian randomization (MR).

**Methods:**

Single nucleotide polymorphisms (SNPs) for iron status were identified from a genome-wide association study (GWAS) on 48,972 individuals. The outcome data came from the largest ALS GWAS to date (20,806 cases; 59,804 controls). We conducted conservative analyses (using SNPs with concordant change of biomarkers of iron status) and liberal analyses (using SNPs associated with at least one of the biomarkers of iron status), with inverse variance weighted (IVW) method as the main analysis. We then performed sensitivity analyses including weighted median, MR-Egger and MR-pleiotropy residual sum and outlier, as well as leave-one-out analysis to detect pleiotropy.

**Results:**

In the conservative analyses, we found no evidence of association between four biomarkers of iron status and ALS using IVW method with odds ratio (OR) 1.00 [95% confidence interval (CI): 0.90–1.11] per standard deviation (SD) increase in iron, 0.96 (95% CI: 0.77–1.21) in ferritin, 0.99 (95% CI: 0.92–1.07) in transferrin saturation, and 1.04 (95% CI: 0.93–1.16) in transferrin. Findings from liberal analyses were similar, and sensitivity analyses suggested no pleiotropy detected (all *p* > 0.05).

**Conclusion:**

Our findings suggest no causal effect between iron status and risk of ALS. Efforts to change the iron status to decrease ALS incidence might be impractical.

## Introduction

Amyotrophic lateral sclerosis (ALS) is a fatal neurodegenerative disorder characterized by progressive degeneration of upper and lower motor neurons (van Es et al., 2017). Clinical epidemiological studies reported that ALS is more common in Caucasian ethnicities and in the male gender (Verde and Ticozzi, 2016). It has been estimated that the number of ALS cases would increase by nearly 70% in the next 25 years across the globe, causing a large potential socioeconomic and health burden in the coming years (Arthur et al., 2016). Therefore, it is critical to identify modifiable risk factors for ALS.

Iron plays key roles in various biological processes such as oxygen delivery, mitochondrial functions, neurotransmitter biosynthesis, and myelin formation (Thirupathi and Chang, 2019). However, an excess of free iron is also potentially toxic. Increased iron status has been shown to be associated with neurodegenerative disorders (NDs) such as Alzheimer’s disease (AD), Parkinson’s disease (PD), and ALS (Oshiro et al., 2011). In contrast, some observational studies suggested a negative association of iron status with such NDs, and some found no significant difference in iron status between individuals with and without NDs (Ahmed and Santosh, 2010; Madenci et al., 2012). However, causal inferences drawn from such observational studies were limited by reverse causality and residual confounding (Boyko, 2013). Mendelian randomization (MR) has been applied to determine the specific causal relationships, and a protective effect of increased iron status on PD has been confirmed by a recent MR study (Pichler et al., 2013; Burgess et al., 2015).

It has been suggested that increased iron status might be a risk factor for ALS. Several studies have tended to show a positive association of iron status with ALS incidence (Nadjar et al., 2012; Veyrat-Durebex et al., 2014; Sun et al., 2019). If the iron status is the risk factor of ALS incidence, then modulating iron levels could be a novel way for ALS prevention. There is still no randomized control trial (RCT) to determine the causal relationship of iron status with ALS. In this study, we used MR design to comprehensively investigate whether there is a causal effect of iron status on ALS incidence. The fundamental principle of MR study is using genetic variants as instrumental variants robustly associated with potential risk factors to estimate causality on diseases of interest (Smith et al., 2008). Therefore, confounding is less likely because genetic variants are randomly assigned at conception. Besides, genetic variants are allocated before disease onset, and genotypes are not modifiable by diseases, thus making reverse causality less likely. Furthermore, utilizing summary-level data from genome-wide association studies (GWAS) for two-sample MR analyses provides larger statistical power (Burgess et al., 2015).

In this study, we performed a two-sample MR study to explore the potential causality between iron status and incidence of ALS.

## Materials and Methods

**FIGURE 1:**
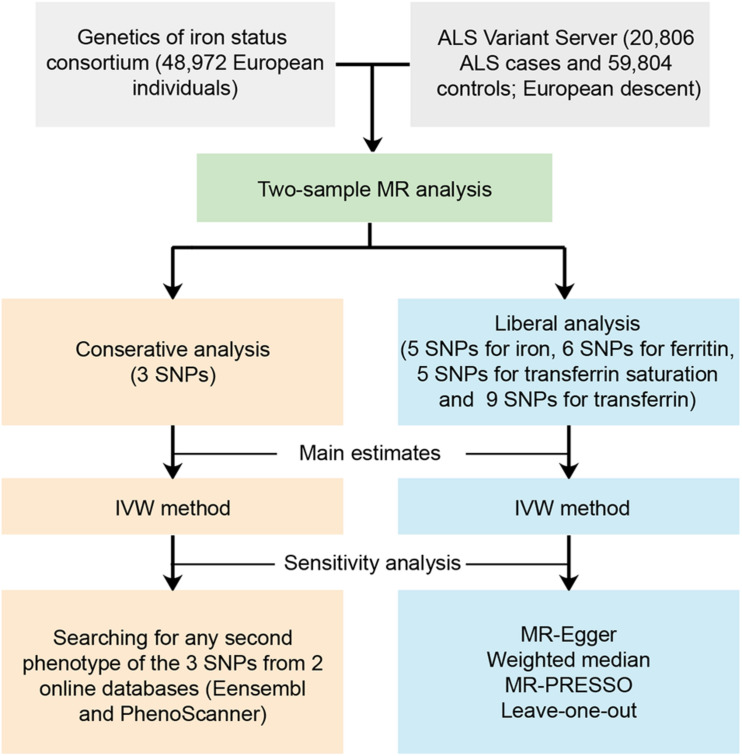
Workflow of the Mendelian randomization study investigating the causal effect of iron status on risk of amyotrophic lateral sclerosis. ALS, amyotrophic lateral sclerosis; SNP, single nucleotide polymorphisms; MR, Mendelian randomization; IVW, inverse variance weighted; MR-PRESSO, Mendelian randomization pleiotropy residual sum and outlier.

We performed a two-sample MR study to investigate the causal effect of iron status on the risk of ALS. Summary data were obtained from GWAS consortia studies. An overview of the study design is presented in Figure 1.

### Instrumental Variables Selection

To ensure the validity of the instrumental variables included for MR analyses, the instruments selected for exposure (iron status) should meet the following criteria: (van Es et al., 2017) single nucleotide polymorphisms (SNPs) robustly associated with exposure at the threshold of genome-wide significance (*p* < 5 × 10^–8^); (Verde and Ticozzi, 2016) all SNPs should be in linkage equilibrium (pairwise *r*^2^ ≤ 0.01); and (Arthur et al., 2016) *F* statistic above 10 was required for sufficient strength to limit the bias from weak instrumental variables (Pierce et al., 2011). To calculate *F* statistic, we used: R^2^×(N−k−1)/[(1−R^2^)×k], where N is the sample size of GWAS for iron status, *k* is the number of SNPs, and R^2^ is the proportion of the variability of iron status explained by each SNP. Specifically, R^2^ was calculated using the formula: 2×beta^2^×(1−EAF)×EAF, where EAF is the effect allele frequency and beta is the estimate of the genetic effect of each SNP on iron status (Pierce et al., 2011). Exposure-associated SNPs were extracted from outcome data (ALS). All the GWAS consortia used in our MR analyses were restricted to European descents to minimize the potential bias owing to population heterogeneity.

### SNPs for Iron Status

The largest GWAS analysis including 11 discovery cohorts and eight replication cohorts performed by the Genetics of Iron Status (GIS) consortium was used to obtain summary-level data on the association between SNPs and iron status (Supplementary Table 1) (Benyamin et al., 2014). A total of 48,972 European subjects (46.9% for male participants) were involved in the meta-analysis. Specifically, biomarkers of iron status include serum iron, ferritin (log-transformed), transferrin saturation, and transferrin. The association of SNPs with these biomarkers was estimated after adjusting for covariates including age, principal component scores, and other study-specific covariates.

The meta-analysis comprising 19 cohorts by the GIS consortium identified 12 SNPs related to the biomarkers of systemic iron status at genome-wide significance (*p* < 5 × 10^–8^) (Table 1) and no linkage disequilibrium (LD) among them (all pairwise *r*^2^ ≤ 0.01 from^1^) (Benyamin et al., 2014). F statistic for individual SNP ranged from 39 to 3,340, making bias from the inclusion of weak instrumental unlikely (Huang et al., 2019).

**TABLE 1:** Association estimates for SNPs associated with biomarkers of iron status at genome-wide significance (*p* < 5 × 10*^–^*^8^) identified from GIS consortium.

CHR	SNP (position, build 37)	Nearest gene: region (Build 37)	EA/OA	EAF	Beta (SE)			
					Iron	Ferritin (log)	Transferrin saturation	Transferrin
6	rs1799945* (26,091,179)	*HFE:* 26,087,509–26,098,571	C/G	0.85	−0.19 (0.01)	−0.07 (0.01)	−0.23 (0.01)	0.11 (0.01)
6	rs1800562* (26,093,141)	*HFE:* 26,087,509–26,098,571	A/G	0.07	0.33 (0.02)	0.20 (0.02)	0.58 (0.02)	−0.48 (0.02)
22	rs855791* (37,462,936)	*TMPRSS6:* 37,461,476–37,505,603	A/G	0.45	−0.18 (0.01)	−0.06 (0.01)	−0.19 (0.01)	0.04 (0.01)
3	rs8177240 (133,477,701)	*TF:* 133,464,800–133,497,850	T/G	0.67	−0.07 (0.01)		0.10 (0.01)	−0.38 (0.01)
7	rs7385804 (100,235,970)	*TFR2:* 100,218,039–100,240,402	A/C	0.62	0.06 (0.01)		0.05 (0.01)	
9	rs651007 (136,153,875)	*ABO:* 136,125,788–136,150,617	T/C	0.20		−0.05 (0.01)		
17	rs411988 (56,709,034)	*TEX14:* 56,634,039–56,769,416	A/G	0.46		−0.04 (0.01)		
2	rs744653 (190,378,750)	*WDR75:* 190,306,159–190,340,291 *SLC40A1:* 190,425,305–190,448,484	T/C	0.85		−0.09 (0.01)		0.07 (0.01)
3	rs9990333 (195,827,205)	*TFRC:* 195,754,054–195,809,060	T/C	0.46				0.06 (0.01)
8	rs4921915 (18,272,466)	*NAT2:* 18,248,755–18,258,728	A/G	0.78				0.08 (0.01)
11	rs6486121 (13,355,770)	*ARNTL:* 13,298,199–13,408,813	T/C	0.63				−0.05 (0.01)
11	rs174577 (61,604,814)	*FADS2:* 61,560,452–61,634,826	A/C	0.33				0.06 (0.01)

*indicates SNPs used for conservative analyses. CHR, chromosome; SNP, single nucleotide polymorphism; GIS, Genetic of Iron Status; SE, standard error; EA, effect allele; OA, other allele; EAF, effect allele frequency.

Using these 12 SNPs, we conducted conservative analyses and liberal analyses. For conservative analyses, only three SNPs were included for MR analyses. Increased concentration of iron and ferritin, increased transferrin saturation, and decreased transferrin are associated with increased systemic iron status (Wish, 2006). Therefore, SNPs used to genetically predict systemic iron status were expected to have a concordant change of four aforementioned biomarkers. Among these 12 SNPs, 3 SNPs (rs1800562, rs1799945, and rs855791) were related to concordant change of four biomarkers at genome-wide significance, which indicated a consistent association with iron status and thus were included for conservative analyses. To test the robustness of our findings to potential pleiotropy, we performed liberal analyses, in which we relaxed the instrumental variables selection criteria to employ more SNPs available for analyses. Specifically, we included all SNPs associated with at least one of four biomarkers at a threshold of *p* < 5 × 10^–8^ for liberal analyses.

### GWAS Data for ALS

Genome-wide association studies summary-level data for ALS were obtained from ALS Variant Server (AVS), which was also carried out on a European population (Nicolas et al., 2018). Specifically, Nicolas et al. conducted a GWAS analysis on 44,558 European descents [8,229 ALS cases (female, 41.7%; mean age, 59.8 ± 12.3) and 36,329 control subjects (female, 69.6%; mean age, 63.4 ± 13.9)]. Then, they incorporated their data into meta-analysis with a previously published ALS GWAS conducted by van Rheenen et al. (2016) (12,577 ALS cases and 23,475 control samples), thus contributing to a large-scale GWAS involving 80,610 European descents (20,806 ALS cases and 59,804 control samples). Detailed dataset information is presented in Supplementary Table 1. All cases included in the analyses were diagnosed with ALS according to EI Escorial criteria (Brooks, 1994). Imputation and quality control were conducted in the ALS GWAS meta-analysis, and nearly 10 million genotyped and imputed variants are available. Twelve SNPs associated with iron status were all extracted from ALS GWAS data.

### MR Estimates

For both conservative and liberal analyses, inverse variance weighted (IVW) method was used for the main MR estimates to detect the effect of each measure of iron status on the risk of ALS. This method assumes that all instrumental variants are valid based on the MR assumptions and combines the Wald ratio estimates of the causal effect obtained from different SNPs to provide a consistent estimate of the causal effect of the exposure on the outcome (Pierce and Burgess, 2013). The *a priori* power calculation was also conducted for the IVW model. Specifically, we calculated the detectable effects of iron status on the risk of ALS at the threshold of 80% power using an online calculator^2^ (Brion et al., 2013).

### Sensitivity Analyses

Mendelian randomization design is based on the assumption that instrumental variables affect the outcome only through their effect on the exposure of interest (iron status in this study), and instrumental variables should be independent of any confounders (Boef et al., 2015). Violation of this assumption may introduce bias to MR results. Therefore, we conducted sensitivity analyses to detect and adjust for any potential heterogeneity and pleiotropy. However, methods for sensitivity analyses require an adequate number (at least five SNPs) of instrumental variables to catch substantial variance, and thus, such methods are not applicable to conservative analyses (only three SNPs) in this study (Ong and MacGregor, 2019). Despite this, we searched for any second phenotypes of the three SNPs included in conservative analyses from two online databases (Ensembl^3^; PhenoScanner^4^) to detect any potential functional pleiotropy and then removed the SNPs with possible pleiotropic effects from the IVW model (Burgess and Thompson, 2015).

The liberal analyses mentioned above introduced more SNPs by relaxing selecting criteria, making statistical sensitivity analyses applicable. Therefore, for the liberal analyses, except for the IVW method, we also estimated the causal effect using the weighted median (WM) method and MR-Egger regression, which were more robust to the inclusion of instrumental variants with potential pleiotropy at the cost of decreased statistical power. WM method allows for 50% of the instrumental variables to be invalid, and MR-Egger method provides an intercept as an indicator of average pleiotropic bias (Bowden et al., 2015; Burgess et al., 2017). Besides, Mendelian randomization pleiotropy residual sum and outlier (MR-PRESSO) test was performed to detect outliers with potential horizontal pleiotropy. MR-PRESSO estimates both SNP-level and global heterogeneity to detect horizontal pleiotropy, and the outlier test compares expected and observed distributions of individual variants to identify outlier variants. If any of the outlier variants are detected, they would be discarded to obtain an unbiased causal estimate from an outlier-corrected MR analysis (Verbanck et al., 2018). Finally, leave-one-out analysis was also performed to evaluate whether the MR estimates were driven strongly by an single SNP.

All the analyses were performed by the Two-Sample MR package (version 0.5.4) and MR-PRESSO package (version 1.0) of the R program (version 4.0.0).

## Results

Three SNPs were used as instrumental variables with concordant change of four biomarkers of iron status for conservative analyses. For liberal analyses, we used a larger SNP group for iron (five SNPs), ferritin (six SNPs), transferrin saturation (five SNPs), and transferrin (nine SNPs) (Table 1). By retrieving the GAWS summary data of ALS, 12 iron status-related SNPs were all extracted (Table 2).

**TABLE 2:** Association estimates of SNPs included with ALS from AVS GWAS summary data.

SNP	EA/OA	EAF	Beta	SE	*P*
rs1800562	A/G	0.062	0.02	0.03	0.45
rs1799945	C/G	0.849	0.01	0.02	0.64
rs855791	A/G	0.433	−0.01	0.01	0.33
rs744653	T/C	0.859	−0.02	0.02	0.39
rs8177240	T/G	0.664	0.02	0.01	0.16
rs9990333	T/C	0.459	0.00	0.01	0.75
rs7385804	A/C	0.634	0.03	0.01	0.06
rs4921915	A/G	0.773	−0.01	0.02	0.63
rs651007	T/C	0.215	0.03	0.02	0.05
rs6486121	T/C	0.522	0.00	0.01	0.79
rs174577	A/C	0.340	−0.00	0.01	0.94
rs411988	A/G	0.510	−0.02	0.01	0.23

*SNP, single nucleotide polymorphism; ALS, amyotrophic lateral sclerosis; AVS, ALS variant server; GWAS, genome-wide association study; EA, effect allele; OA, other allele; EAF, effect allele frequency; SE, standard error.*

**FIGURE 2:**
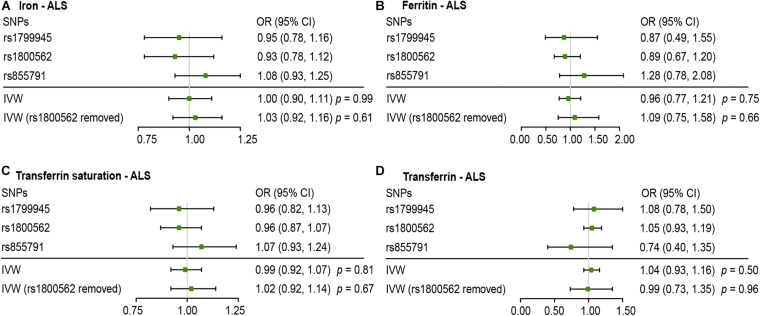
Mendelian randomization (MR) estimates for each of the four biomarkers of iron status in conservative analyses. Panels **(A–D)** respectively indicate the causal estimates of iron, ferritin, transferrin saturation and transferrin on amyotrophic lateral sclerosis (ALS). SNPs, single nucleotide polymorphisms; OR, odds ratio; CI, confidence interval; IVW, inverse variance weighted.

**FIGURE 3:**
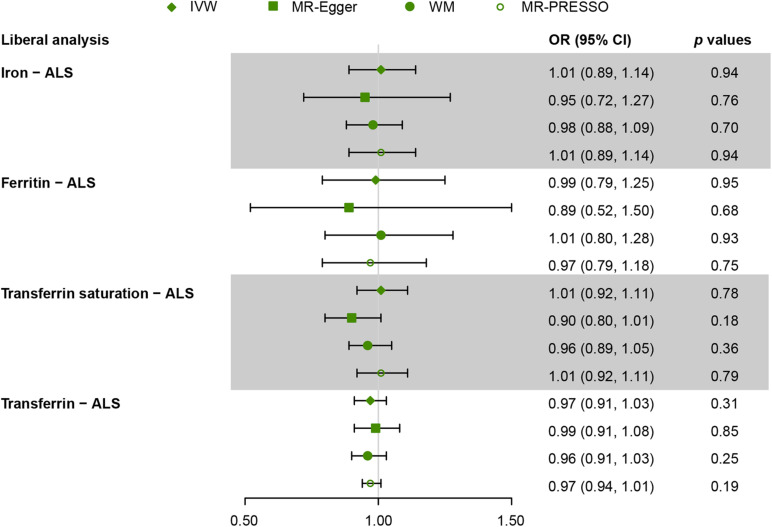
Mendelian randomization (MR) estimates for each of the four biomarkers of iron status in liberal analyses. OR, odds ratio; CI, confidence interval; ALS, amyotrophic lateral sclerosis; IVW, inverse variance weighted; MR-Egger, Mendelian randomization Egger regression method; WM, weighted median; MR-PRESSO, Mendelian randomization pleiotropy residual sum and outlier.

Figures 2, 3 present all the MR results for both conservative and liberal analyses estimating causal effects of iron status on the risk of ALS. The estimates are presented as odds ratios (ORs) for ALS per standard deviation (SD) increase in each biomarker of iron status. Expected ORs achieving at least 80% statistical power are presented in Supplementary Table 2.

For conservative analyses, we found that iron [OR, 1.00; 95% confidence interval (CI), 0.90–1.11; *p* = 0.99), log-transformed ferritin (OR, 0.96; 95% CI, 0.77–1.21; *p* = 0.75), transferrin saturation (OR, 0.99; 95% CI, 0.92–1.07, *p* = 0.81), and transferrin (OR, 1.04; 95% CI, 0.93–1.16, *p* = 0.50) were not causally associated with ALS incidence using IVW method. No individual SNP suggested a risk effect on ALS. In the Ensembl database and PhenoScanner database, we found iron-status-raising allele in rs1800562 was associated with a lower low-density lipoprotein (LDL). MR estimates after removing rs1800562 remained null (Figure 2).

For liberal analyses, the main MR results using the IVW method were similar to the conservative analyses. MR estimates derived from the other three methods, including MR-Egger, WM, and MR-PRESSO, were similar to the IVW method (Figure 3). Intercepts derived from MR-Egger method indicated that any potential pleiotropy of single SNP was balanced, making bias to the results unlikely (for iron, intercept = 0.009, *p* = 0.70; for ferritin, intercept = 0.009, *p* = 0.66; for transferrin saturation, intercept = 0.03, *p* = 0.09; for transferrin, intercept = −0.006, *p* = 0.45). MR-PRESSO identified no outlier variants (for iron, *p* = 0.18; for ferritin, *p* = 0.33; for transferrin saturation, *p* = 0.16; for transferrin, *p* = 0.70). Leave-one-out analysis was conducted by removing individual SNP from instruments in turn, and the estimated effects derived from the IVW model remained null (Supplementary Figure 1).

## Discussion

In this study, we performed a two-sample MR analysis to comprehensively investigate the causal effect of iron status on ALS using the largest GWAS summary-level data publicly available to date. Our results found no causal effect of iron status on the risk of ALS, suggesting no evidence for iron status as a prospective target for the prevention of ALS.

The role of iron metabolism in NDs has long been noted. A body of evidence from animal models suggested abnormal iron homeostasis in ALS (Jeong et al., 2009; Wang et al., 2011). Besides, mutations in superoxide dismutase 1 (SOD1) have been reported among some of the ALS cases (Yoshida et al., 2010). Dysfunction of SOD1 breaks the dynamic equilibrium between ions and free radicals, leading to metabolic disturbance (including iron metabolism) (Oshiro et al., 2011). Oxidative stress and the following neurotoxicity induced by iron dyshomeostasis have been considered as one of the key pathways contributing to the development of NDs, including ALS (Oshiro et al., 2011). As such, efforts were further taken to reveal the association between iron status and ALS risk in clinical studies, which have tended to provide evidence that increased ALS incidence is associated with increased iron status (Nadjar et al., 2012; Veyrat-Durebex et al., 2014; Sun et al., 2019). However, such findings were derived from observational studies, which could not distinguish between increased iron status causing ALS or ALS causing an increased iron status (reverse causality). Besides, observative results are likely to be biased by unrevealed risk factors. While a majority of studies have adjusted for potential confounders like gender, age, body mass index (BMI), and smoking and drinking habits, there was still residual confounding present such as dietary habits, military service, and organic solvents exposure, which were challenging to be adjusted for completely (Morozova et al., 2008; Fang et al., 2009; Bryan et al., 2016). Furthermore, exposure of interest and outcome may sometimes be the common consequence of another exposure. For example, viral infection was reported to induce iron overload (Drakesmith and Prentice, 2008). A previous study suggested viral infection, and subsequent immune response might induce ALS (Cermelli et al., 2003). Therefore, increased iron status and ALS incidence might be commonly induced by a viral infection, which should be further confirmed by future studies.

Owing to the limitations of previous observational studies mentioned above, the causal effect of iron status on ALS remained unknown. RCTs are widely accepted for exploring causality but at a high cost of time, money, labor, and material resources. It is impossible to explore all these causal relationships through RCTs. Instead, collecting evidence to comprehensively reveal these causal relationships through other study designs like MR approach is more practical. The present study used MR approach to comprehensively investigate the potential causal effect of iron status on ALS risk.

As MR study may carry the risk of pleiotropy, we adopted various strategies to detect and correct the potential pleiotropy. For the conservative analyses, we searched online for the second phenotypes of the three SNPs. We found that rs1800562 (effect allele: A) was also associated with decreased serum level of low-density lipoprotein (LDL) at genome-wide significance. A recent MR study has confirmed a causal effect of increased LDL on ALS incidence (Zeng and Zhou, 2019). Consistent with the hypothesis of some bias owing to pleiotropy, directions of MR estimate after removing rs1800562 were reversed, but the association remained null (Figure 2). Thus, our results of conservative analyses were unlikely to be severely affected by this pleiotropy.

We further increased the number of instrumental variables for analyses (liberal analyses) by relaxing our SNPs selecting criteria to test whether our results were robust to potential pleiotropy. The results of the main MR estimate using IVW in liberal analyses were similar to the conservative analyses. The MR estimates derived from the WM method and MR-Egger regression remained null. Besides, the MR-Egger intercepts suggested no average pleiotropic bias. In addition, MR-PRESSO was conducted to detect and correct for horizontal pleiotropic outliers. No outliers were identified in our study, and MR estimates derived from MR-PRESSO remained null. Furthermore, leave-one-out MR estimates suggested no individual SNP driving the pooled IVW estimates. Based on these thorough and comprehensive detections of potential pleiotropy, the overall conclusions of our study were less likely to be strongly affected by bias.

Our work has several important strengths. Compared to the observational study, using genetically predicted phenotype as exposure of interest in MR study makes reverse causality and confounding bias less likely. Besides, the present study used GWAS summary data obtained from the largest scale of meta-studies to date. In the conservative analyses, rs1800562 [*HFE* (*C282Y*)], rs1799945 [*HFE* (*H63D*)], and rs855791 [*TMPRSS6* (*V736A*)] were obtained from GIS consortium (totally 48,972 participants) (Benyamin et al., 2014). Previous studies have tended to provide conflicting evidence of the association between the *C282Y* and *H63D* polymorphisms in the *HFE* gene and ALS risk (Yen et al., 2004; Goodall et al., 2005; Li et al., 2014). However, all these studies were relatively small and were underpowered to detect modest genetic effects ALS risk. In our study, associations of these polymorphisms with ALS were extracted from the largest ALS case–control sample with genetic data meta-analyzed (a total of 20,806 ALS patients and 59,804 controls) to date (Nicolas et al., 2018). Hence, it is less likely to violate the assumptions of the MR study (Boef et al., 2015). Last, GWAS data utilized in the present study were limited in European descents to reduce possible bias from population heterogeneity.

Several limitations should be considered in our study. First, all participants included in our study were restricted to European ancestry in order to reduce possible bias attributable to ethnic differences. However, whether our findings are universal to other populations still needs to be confirmed. Second, using GWAS summary-level data makes it hard to conduct stratified analyses by age, gender, and other features of interest. Furthermore, although liberal analyses increased instrumental variants to offer more power and allowed more methods for detecting and correcting pleiotropy, there might be residual confounding because other certain functions of iron-status-related SNPs used in our study remained unknown to date. Third, survival bias should also be considered in our study. A recent MR study suggested that higher system iron status might reduce lifespan (Daghlas and Gill, 2020). Since ALS is a late-onset disease, higher iron levels may be expected to increase death risk prior to living to the typical age of onset of ALS. As such, the causal inference of iron with ALS may be influenced by survival bias. This bias generally limits the interpretation of results from MR investigations of late-onset neurodegenerative disease (Noyce et al., 2017). Fourth, although our study found no causal effect of iron status on ALS incidence, it did not suggest no association of iron status with ALS prognosis. Actually, factors that affect the incidence and prognosis of a disease can be different, and our study tended to provide evidence that iron status might not be a promising target for ALS prevention (Paternoster et al., 2017). Finally, as shown in Supplementary Table 2, the expected ORs at 80% power indicated that the present IVW estimates had limited statistical power, and hence, further work is needed to verify our results when a larger scale of GWAS is available in the future.

In conclusion, our study found no causal effect of iron status on ALS risk. Association between increased iron status and a higher ALS incidence obtained from previous studies might be partly owing to unmeasured confounders or reverse causality. Efforts to change iron concentrations to decrease the risk of ALS might be impractical.

## Data Availability Statement

The original contributions presented in the study are included in the article/Supplementary Material, further inquiries can be directed to the corresponding author/s.

## Ethics Statement

We used summary-level data publicly available. Appropriate patient consent and ethical approval were obtained in the original studies.

## Author Contributions

JC, XC, HW, and YP designed the manuscript. JC, XC, and HW were performed the statistical analyses. JC, XC, and ZW were written the first draft of the manuscript. All authors contributed to the interpretation of data and commented the previous versions of the manuscript. All authors read and approved the final version of the manuscript.

## Conflict of Interest

The authors declare that the research was conducted in the absence of any commercial or financial relationships that could be construed as a potential conflict of interest.

## Footnotes

1 https://ldlink.nci.nih.gov/?tab=home

2 https://shiny.cnsgenomics.com/mRnd/

3 https://uswest.ensembl.org/index.html

4 http://www.phenoscanner.medschl.cam.ac.uk/

## Supplementary Material

Supplementary Table 1 Detailed information for genome-wide association studies (GWAS) involved in the present Mendelian randomization study.

Supplementary Table 2 Detectable causal effects of iron status on amyotrophic lateral sclerosis (ALS) incidence at 80% statistical power in inverse variance weighted (IVW) model.
