# Supplementary material for: Iron Status May Not Affect Amyotrophic Lateral Sclerosis: A Mendelian Randomization Study
Source: Front Genet. 2021 Mar 4;12:617245. doi: 10.3389/fgene.2021.617245 (PMC7969891; doi:10.3389/fgene.2021.617245)
Supplement: Supplementary Table 1 — Detailed information for genome-wide association studies (GWAS) involved in the present Mendelian randomization study. [file Data_Sheet_1.PDF]

Supplementary Material

Figure S1. Leave-one-out analysis for each of the 4 biomarkers of iron status.

Table S1. Detailed information for genome-wide association studies (GWAS) involved in the present Mendelian randomization study.

Table S2. Detectable causal effects of iron status on amyotrophic lateral sclerosis (ALS) incidence at 80% statistical power in inverse variance weighted (IVW) model.

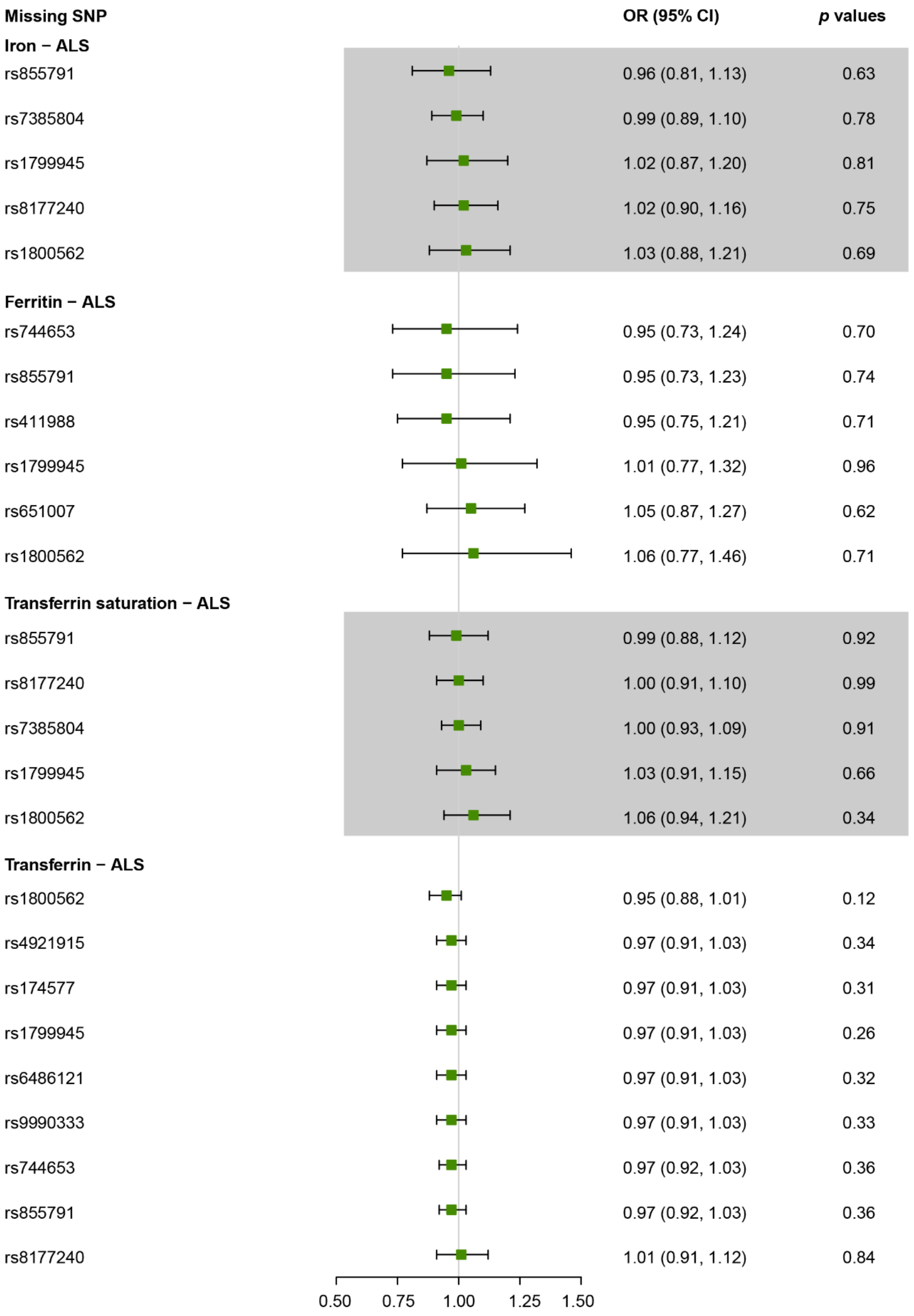

Figure S1. Leave-one-out analysis for each of the 4 biomarkers of iron status. SNP, single nucleotide polymorphism; OR, odds ratio; CI, confidence interval; ALS, amyotrophic lateral sclerosis.

Table S1. Detailed information for genome-wide association studies (GWAS) involved in the present Mendelian randomization study.

Phenotypes	Consortium	Ancestry	Sample size	Nations and cohorts
Iron status	Genetics of Iron Status (GIS)	European	48,972 individuals	**Australia:** Australia-Adult; Australia-Adolescent; Busselton Health Study<br>**Estonia:** Estonia (original); Estonia (replication)<br>**Germany:** KORA F3; KORA F4<br>**Italy:** Val Borbera; Micros/EURAC; InCHIANTI; SardiNIA<br>**The Netherlands:** Nikmegen Biomedial Study; ERF/Rotterdam; PREVEND<br>**United Kingdom:** Cambridge; FENLAND<br>**Switzerland:** CoLAUS<br>**Multi-nations:** INTERACT
Amyotrophic lateral sclerosis (ALS)	ALS variant server (AVS)	European	20,806 ALS cases; 59,804 control samples	**Belgium:** BE1~BE2<br>**Finland:** FIN1~FIN4<br>**France:** FR1~FR3<br>**Germany:** GER1~GER3<br>**Ireland:** IR1~IR3<br>**Italy:** IT1~IT4<br>**Portugal:** PU1<br>**Spain:** SP1<br>**Sweden:** SW1~SW2<br>**Switzerland:** SWISS1<br>**The Netherlands:** NL1~NL5<br>**United Kingdom:** UK1~UK5<br>**United State:** US1~US7<br>**Multi-nations:** LNG

Table S2. Detectable causal effects of iron status on amyotrophic lateral sclerosis (ALS) incidence at 80% statistical power in inverse variance weighted (IVW) model.

Analysis	Iron status	R^2	OR	Expected effects at 80% power
Conservative analyses	Iron	0.039	1.00	> 1.118 or < 0.890
	Ferritin	0.007	0.96	> 1.286 or < 0.750
	Transferrin saturation	0.073	0.99	> 1.085 or < 0.919
	Transferrin	0.033	1.04	> 1.128 or < 0.880
Liberal analyses	Iron	0.043	1.01	> 1.111 or < 0.894
	Ferritin	0.012	0.99	> 1.219 or < 0.805
	Transferrin saturation	0.079	1.01	> 1.082 or < 0.922
	Transferrin	0.104	0.97	> 1.071 or < 0.931

R^2 indicated the proportion of variability of iron status explained by the single nucleotide polymorphisms (SNPs); OR indicated the odds ratios for ALS risk per standard deviation change of corresponding iron status markers in the present Mendelian randomization IVW model.
